# The Presence of Physical Symptoms in Patients With Tinnitus: International Web-Based Survey

**DOI:** 10.2196/14519

**Published:** 2019-07-30

**Authors:** Sarah Michiels, Stephen Harrison, Markku Vesala, Winfried Schlee

**Affiliations:** 1 Department of Rehabilitation Sciences and Physiotherapy Faculty of Medicine and Health Sciences University of Antwerp Wilrijk Belgium; 2 Department of Otorhinolaryngology Antwerp University Hospital Edegem Belgium; 3 Department of Translational Neurosciences Faculty of Medicine and Health Sciences University of Antwerp Wilrijk Belgium; 4 Tinnitus Hub Ltd Hemsworth United Kingdom; 5 Department of Psychiatry and Psychotherapy University of Regensburg Bezirksklinikum Regensburg Regensburg Germany

**Keywords:** tinnitus, self report, surveys

## Abstract

**Background:**

Tinnitus, or ringing in the ears, is a phantom perception of sound in the absence of overt acoustic stimulation. Many patients indicate that the perception of their tinnitus is not constant and can vary from moment to moment. This tinnitus fluctuation is one of the diagnostic criteria for somatosensory tinnitus (ST), a tinnitus subtype that is influenced by cervical spine or temporomandibular dysfunctions, although various factors have been reported to cause fluctuations in tinnitus, such as stress, anxiety, and physical activity.

**Objective:**

The aim of this study was twofold: (1) to investigate the presence of physical symptoms in a large group of participants with tinnitus and (2) to investigate if these physical symptoms are more frequently present in a subgroup of participants with ST.

**Methods:**

A Web-based survey, questioning the presence of physical symptoms in a convenience sample of participants with tinnitus, was launched on the online forum, Tinnitus Talk, managed by Tinnitus Hub. After a general analysis of the physical symptoms present in our survey population, we further analyzed the group of participants who were diagnosed by a physician (n=1262). This subgroup was divided into 2 groups, one group diagnosed with ST and another group diagnosed with other types of tinnitus.

**Results:**

In total, 6115 participants with a mean age of 54.08 years (SD 13.8) completed the survey. Physical symptoms were frequently present in our sample of participants with tinnitus: 4221 participants (69.02%) reported some form of neck pain, 429 (7.01%) were diagnosed with temporomandibular disorders, 2730 (44.64%) indicated they have bruxism, and between 858 and 1419 (14.03%-23.20%) participants were able to modulate their tinnitus by voluntary movements. ST was diagnosed in 154 out of 1262 (12.20%) participants whose tinnitus cause was diagnosed by a physician. Symptoms referring to the known diagnostic criteria were evidently more present in the ST group than in the non-ST group. Additionally, participants with ST more often indicated a negative effect of a bad night’s sleep (*P*=.01) and light intensity exercise (*P*=.01).

**Conclusions:**

Physical activity and movement (disorders) frequently affect tinnitus severity. Head-neck related symptoms are more frequently reported in the ST group, as is the ability to modulate the tinnitus by head or jaw movements. Additionally, participants with ST more often report fluctuations of their tinnitus and reaction to sleeping difficulties and low intensity exercise.

## Introduction

Tinnitus is the phantom perception of sound in the absence of overt acoustic stimulation [1]. The perception of tinnitus can be tonal or noise-like and is often described as hissing, sizzling, or ringing [2]. If the perception of tinnitus is ongoing for more than 6 months, the condition is considered as *chronic tinnitus*. This occurs in about 10% to 15% of adults [2] and is often related to hearing loss or a noise trauma, where cochlear abnormalities are the initial source and neural changes in the central auditory system maintain the tinnitus [2]. Furthermore, for many, the perception of tinnitus is not constant and can vary from moment to moment [3]. This fluctuation of tinnitus can depend on various factors, such as stress [4], emotional states [5], anxiety [6], depression [6], cervical spine dysfunction [7], and temporomandibular disorders (TMDs) [8], but also, physical activity [9,10] has been reported to influence the perception of tinnitus.

In this study, we were interested in the influence of physical activity on the perception of tinnitus. It has been found that accelerometer-assessed physical activity correlates negatively with tinnitus severity [9,10] and correlates positively with health-related and global quality of life [10]. In contrast, patients in clinical practice often complain about an increase in tinnitus loudness during or immediately after physical activity. Additionally, it is still unclear if these effects are present in all patients with tinnitus.

Furthermore, cervical spine and TMDs can be a risk factor for tinnitus, which is often referred as somatosensory tinnitus (ST) [7,11-13]. In this type of tinnitus, changes in somatosensory afference from the cervical spine or temporomandibular area are causing or changing the tinnitus percept. ST can be diagnosed based on the presence of a series of characteristics of the tinnitus, such as the ability to change the tinnitus by certain movements, and accompanying symptoms, such as neck pain, headache, or jaw pain [12]. However, it is not known if other physical influences are also more common in patients with ST than in patients with other types of tinnitus.

The aim of this study was therefore twofold: first to investigate the presence of physical symptoms in a large group of participants with tinnitus and second to investigate if these physical symptoms are more frequently present in a subgroup of participants with ST.

## Methods

### Survey

A Web-based survey, questioning the presence of physical symptoms in a convenience sample of participants with tinnitus, was launched on the online forum, Tinnitus Talk, managed by Tinnitus Hub, in February 2017. The idea of the survey topic was conceived from talking to patients and moderating the online forum. Questions were designed on consultation with tinnitus researchers and a small pool of the forum’s community and trialed with this group before launch. This was done to make sure that all questions were clear and unambiguous and that no technical issues were present. The final questionnaire consisted of 21 questions that asked for different physical symptoms that can accompany the tinnitus and for a set of tinnitus characteristics. The complete list of questions is displayed in Multimedia Appendix 1.

The survey was advertised on the Tinnitus Hub website and launched as a closed survey, open to all registered users of the online forum, Tinnitus Talk. No incentives were offered to participants. An internet protocol check was used to identify and block potential duplicate entries from the same user. All participants gave informed consent to use their anonymized data. No personal information was collected during the process.

### Data Analysis

#### General Characteristics and Physical Symptoms

Participant characteristics and the presence of physical symptoms in the entire group were analyzed using descriptive statistics and frequencies.

#### Somatosensory Tinnitus

Participants who indicated in question 6 that their tinnitus cause was diagnosed by a physician were separated from the rest of the patients. The group that was diagnosed by a physician was then used to create 2 subgroups: one diagnosed with ST (ST group) and another with other tinnitus diagnoses (non-ST group). Differences between both groups were analyzed using Fisher exact tests in case of dichotomous variables and via independent sample *t* tests for continuous variables. Correction for multiple comparison was made with the Benjamini-Hochberg false discovery rate procedure, using a false discovery rate of 5%. In the Results section, only the corrected Benjamini-Hochberg *P* values are presented. The significance level was set at *P* less than .05.

Only complete questionnaires, without missing data, were used for the analysis. All analyses were performed using IBM SPSS Statistics for Macintosh (version 25.0; IBM Corporation).

## Results

### General Characteristics

A group of 6115 participants (51.60% male participants, 47.80% female participants, and 0.30% transgender participants) with a mean age of 54.08 years (SD 13.8) filled out the Web-based survey (details are presented in Table 1). Participants originated from 62 different countries with the highest percentages from the United Kingdom (25.30%) and the United States (40.40%). In 4157 (67.90%) participants, the primary tinnitus cause corresponded to one of the causes listed in the questionnaire (Table 2) and 1618 (26.50%) participants indicated an unknown cause of their tinnitus. In all other cases, the cause of the tinnitus was marked as *not listed*. The most frequently mentioned causes were noise-induced hearing loss (748 or 12.20%) and noise trauma (624 or 10.20%). ST was identified as the primary cause of the tinnitus in 409 out of 6115 (6.60%) participants and as a secondary cause in 16.30%. Psychological problems, such as stress, anxiety, and depression, were listed as the primary cause in 4.40% of the participants and in 12.90% (996/6115) as a secondary cause.

**Table 1:** Demographics.

Variable		Value
Subjects, n		6115
**Gender, n**		
	Male	3154
	Female	2925
	Transgender	17
Age (years), mean (SD)		54.07 (13.81)
VAS^a^ loudness, mean (SD)		5.62 (1.92)

^a^VAS: visual analogue scale.

**Table 2:** Tinnitus causes (N=6115).

Primary tinnitus cause	Value, n (%)
Age-related hearing loss	250 (4.09)
Allergy to something	28 (0.46)
Barotrauma	65 (1.06)
Dental treatment	51 (0.83)
Ear wax build up	36 (0.59)
Ear wax procedure	69 (1.13)
Eustachian tube dysfunction	108 (1.77)
Head or neck injury	217 (3.55)
Menière’s disease	170 (2.78)
Metabolic disease	55 (0.90)
Noise induced hearing loss	748 (12.23)
Noise trauma	624 (10.20)
Otosclerosis	48 (0.78)
Ototoxic medication	304 (4.97)
Psychological	269 (4.40)
Sudden hearing loss	237 (3.88)
Temporomandibular dysfunction	141 (2.31)
Virus	476 (7.78)
Unknown	1618 (26.46)
Cause not listed	340 (5.56)

The duration of tinnitus ranges from very recent to more than 30 years with an average of 6.26 years (SD 6.81). The average tinnitus loudness over the last week on the visual analogue scale (VAS) was 5.62 (SD 1.92). The average tinnitus annoyance over the last week on the VAS was 5.12 (SD 2.24).

A total of 1763 (62.80%) participants were diagnosed with moderate-to-severe hearing loss and 3976 (65.40%) reported to experience a negative effect on their tinnitus from emotional stress and 3756 (62.00%) from anxiety.

### Presence of Physical Symptoms

In our group of 6115 participants, 4221 (69.00%) pointed out that they regularly get neck pain, although only 649 (10.60%) participants have an actual medical condition related to this neck pain (Multimedia Appendix 2). Only 429 out of 6115 (7.00%) participants were diagnosed with TMDs, although 668 (10.90%) complained about jaw pain, 517 (8.50%) about a tired feeling in the jaw muscles, and 1126 (18.40%) about clicking of the jaw. Additionally, 2730 (44.60%) participants have bruxism and 2325 (38.00%) complain about frequent headache episodes.

Clinically, participants often complain about changes in their tinnitus pitch or loudness, which often makes it hard to keep the tinnitus out of the conscious perception. In our group, 4250 participants (69.50%) report daily fluctuations of their tinnitus and 2732 (44.70%) have fleeting episodes at least once a month. Apart from tinnitus changes after sound exposure (63.50%), tinnitus can also be modulated by different movements of the head, neck, or jaw or by pressure on different parts of the body. In our study, the largest group reported that they could modulate their tinnitus by clenching their teeth (1419 or 23.20%) or pushing their jaw outward (1304 or 21.30%). More details can be found in Multimedia Appendix 3.

With regard to the short-term influence of physical activity on tinnitus, we found that 902 participants (14.80%) reported positive effects of light exercise, whereas only 496 (8.10%) indicated a positive effect of an intense workout on their tinnitus. On the contrary, 1195 (19.50%) complained about a negative influence of an intense workout on their tinnitus, compared with only 574 (9.40%) that complained after light exercise (Multimedia Appendix 4).

### Somatosensory Tinnitus

The cause of the tinnitus was diagnosed by a physician in 1262 participants (20.60%). For the following part of the results, we only used these 1262 participants who were divided into 2 groups based on the diagnosis: one group of participants diagnosed with ST (ST group) and another group with other diagnoses (non-ST group).

In 154 out of 1262 participants (12.20%), ST was diagnosed as either the primary (5.80%) or secondary (11.90%) cause of the tinnitus. The results of the comparison of the ST and non-ST group are shown in Table 3 and Multimedia Appendices 5-7. No significant differences in age and gender were found between both groups (*P*=.61). With regard to the tinnitus pitch, 61 out of 154 (42.40%) participants of the ST group describe their tinnitus as a mixture of tones, compared with 367 out of 1108 (32.10%) in the non-ST group. In the ST group, a significantly higher percentage reported that his or her tinnitus varied during the day. A feeling of fullness in the ears after activity was also more present in the ST group. With regard to the ability to modulate the tinnitus by head or jaw movements, all types of modulation are significantly more often present in the ST group, except for the modulation by pushing the jaw outward.

Head-neck–related symptoms are evidently more frequently reported in the ST group. For instance, participants with ST indicated more often that they suffered from stiff or sore neck muscles and from headache. Similarly, TMD symptoms are more often present in the ST group. Significant differences were found for the presence of bruxism and pain or discomfort in the jaw.

**Table 3:** Presence of diagnostic criteria for somatosensory tinnitus in participants with and without somatosensory tinnitus.

Characteristics referring to DC-ST^a^	ST^b^ group (n=154), %	Non-ST group (n=1108), %	Corrected *P* value
Neck pain from medical condition	25.00	8.00	<.001
Restricted neck movement	27.00	13.00	<.001
Headaches	60.00	33.00	<.001
Bruxism	66.00	44.00	<.001
Tinnitus modulation while pressing jaw	33.00	16.00	<.001
Tinnitus modulation while pushing jaw backward	28.00	16.00	<.001
Tinnitus modulation while looking up	33.00	16.00	<.001
Jaw pain	28.00	9.00	<.001
Jaw blockage	10.00	1.00	<.001
Jaw tired feeling	21.00	7.00	<.001
Tense jaw muscles	36.00	11.00	<.001
Jaw clicking	38.00	14.00	<.001
TMD^c^ diagnosis	33.00	4.00	<.001
Neck pain from bad posture	30.00	19.00	.01
Tinnitus modulation while pushing head forward against resistance	28.00	18.00	.01
Tinnitus changes during the day	81.00	70.00	.01
Neck pain after physical activity	22.00	13.00	.01
Tinnitus modulation while clenching teeth	34.00	24.00	.01
Tinnitus modulation while pushing jaw outwards	30.00	23.00	.08
Neck pain from lying in bed	24.00	20.00	.33

^a^DC-ST: diagnostic criteria for somatosensory tinnitus.

^b^ST: somatosensory tinnitus

^c^TMD: temporomandibular disorder.

Additionally, participants diagnosed with ST more often reported a negative effect of a bad night’s sleep on their tinnitus. Negative effects of an intense workout and moderate exercise were present in 265 and 239 out of 1262 (20.00%-25.00%) participants, respectively, but no significant differences were found between both groups. The negative influence of light exercises, on the contrary, was more often present in the ST group.

## Discussion

### Principal Findings

The aim of this study was twofold: first to investigate the presence of physical symptoms in a large group of participants with tinnitus and second to investigate if these physical symptoms are more frequently present in a subgroup of participants with ST.

In general, our study population is similar to tinnitus populations in other studies with regard to age and average tinnitus loudness and annoyance [7,14-16].

In a study sample of 6115 participants, we found that physical symptoms are frequently present in participants with tinnitus: 69.00% of the tinnitus participants reported some form of neck pain. This number falls within the range of lifetime prevalence numbers for nonspecific neck pain [17], but it is still higher than the average lifetime prevalence of 48.50%. For comparison, the occurrence for headache in 38.00% of the cases corresponds roughly to the prevalence of an active headache disorder in the general population [18].

In our study, 7.00% of the participants were diagnosed with TMD and 10.90% complained about jaw pain, which corresponds to the prevalence of jaw pain in the general adult population [19]. However, we would have expected this prevalence to be higher, as tinnitus is a very common symptom in participants with TMD. In the literature, tinnitus prevalence between 30.40% and 64.00% are reported in the TMD population [8,20,21]. Jaw muscle tightness and jaw clicking, which are symptoms of TMD, were reported by 14.80% and 18.40% of our study population, respectively, and bruxism, a parafunction that can lead to TMD, was present in 44.60%. One explanation for the low number of TMD diagnosis in our study might be that, not everyone with TMD symptoms visited a health care provider for his or her symptoms, resulting in an underestimation of the actual number of participants with TMDs. Another explanation might be that the studies reporting the strong association between tinnitus and TMDs are always situated in a primary TMD population. Therefore, it is hard to tell if all these participants with TMDs who also perceive tinnitus are actually bothered by their tinnitus. Our results from a primary tinnitus population, where the prevalence of TMDs is not higher than in the general population, strongly suggest this statement.

Daily fluctuations of the tinnitus pitch or loudness are reported by 69.50% of our participants. These fluctuations are known to be very typical in patients with ST and they are included in the list of diagnostic criteria for ST [12]. Our study also pointed out that fluctuations are more often present in the ST group but the large percentage of participants reporting daily fluctuations in the non-ST group, however, suggests that this item should not be used as a single criterion for diagnosing ST. More specifically, 14.00% to 23.10% of our participants were able to modulate their tinnitus by one of the listed voluntary movements. Additionally, tinnitus modulation was more often reported in the ST group than in the non-ST group, although it must be noted that only 28.00% to 34.00% of the participants in the ST group reported tinnitus modulation. Therefore, the absence of tinnitus modulation should never be the only reason to exclude ST diagnosis [12].

As mentioned in the Introduction section, physical activity can positively influence tinnitus, as especially moderate physical activity reduces stress levels [22]. In our population, only 11.50% reported a positive effect of moderate exercise, whereas 18.30% contrarily reported a negative influence. Generally, our results show that the higher the intensity of the workout, the higher the percentage of participants who perceive a negative influence and the lower the percentage who experience a positive effect. These findings are in contrast with the findings of Carpenter-Thompson et al [10], who stated that higher levels of physical activity were significantly associated with lower levels of tinnitus severity on the Tinnitus Functional Index. This contradiction can be explained by the temporary increase in blood pressure associated with higher physical activity and the fact that tinnitus is associated with arterial hypertension [23-25]. On the contrary, physical activity shows positive long-term effects on arterial hypertension [26]. Despite the large sample size, our study is based on retrospective, self-reported data, which might have influenced the results. More prospective research is needed to investigate the relationship of physical exercise and its impact on tinnitus.

Of the 6115 included patients, 22.90% indicated that they had some form of ST. This corresponds to the 26.50% prevalence of ST in the tinnitus clinic of the Antwerp University Hospital (unpublished data) and to data from a cohort study of Ward et al [27] in the United Kingdom. Other studies have mentioned higher prevalences of ST, ranging from 43.00% to 83.00% depending on the used diagnostic criteria [7,28]. In 2018, a new set of diagnostic criteria for ST was agreed on by an international group of tinnitus experts [12]. As these criteria were not yet available when the survey was launched, it is not entirely clear how the ST diagnosis was made in our survey and some underdiagnosing might be present.

Items such as the patient’s ability to modulate the tinnitus are often used for diagnosing ST, which reflects clearly in the higher percentages of modulation in the ST group, but it must be noted that tinnitus modulation is also present in 16.00% to 24.00% of the non-ST group. Other criteria that might be used for diagnosing ST, such as the presence of neck pain, headache, or temporomandibular joint problems, are similarly more often present in the ST group although small percentages are present in the non-ST group. One very specific criterion from the new list, *Tinnitus is reported to vary*, was also more often present in the ST group (81.00%), but it is also present in 70.00% of the non-ST group. Some caution with the use of this criterion is therefore needed. Additionally, patients in the ST group more often describe their tinnitus as a mixture of tones.

Interestingly we found a significantly higher percentage of participants in the ST group who complained about the “feeling of fullness in the ears after activity.” This symptom has, to our knowledge, never been described as typical for patients with ST. On the contrary, fullness in the ears has been described as a common symptom in patients with TMDs [29]. Further analysis of our data showed that the participants who indicate they have a “feeling of fullness in the ears after activity” significantly more often suffer from pain or dysfunction of the jaw.

As mentioned earlier, physical activity, and especially the higher intensity physical activity, can have a negative effect on tinnitus severity. No significant differences were found between ST and non-ST groups for the negative effect of high or moderate intensity physical activity. However, we did find significantly more participants in the ST group indicating a negative effect of light intensity exercise.

Finally, participants in our ST group reported significantly more often a negative effect of a bad night’s sleep compared with the non-ST group. These findings are logical if we consider that not only tinnitus is affected by sleeping difficulties but also neck and jaw pain that are causing the somatosensory influence in the ST group [30,31].

### Limitations

Our study has 1 major limitation: the self-reported nature of the data, which is of course inextricably linked to survey-based studies. Patients might, for instance, not always remember the exact diagnosis the physician made, especially when the consult was years ago. Additionally, some items, such as “the effect of exercise on tinnitus severity” might be unknown in a sedentary population, resulting in an underestimation of the presence of this symptom. Additionally, the survey was advertised as investigating physical links in tinnitus. This might have introduced some bias, as people who experienced some physical influence on their tinnitus in the past would be extra motivated to participate. However, the large sample already corrects largely for this potential bias. Despite these limitations, our study was able to identify a set of physical symptoms that are often present in participants with tinnitus. Future studies should aim to investigate these symptoms in a more controlled and detailed way to draw any definitive conclusions.

### Conclusions

Physical activity and movement (disorders) frequently affect tinnitus severity. Head-neck–related symptoms are more frequently reported in the ST group, as is the ability to modulate the tinnitus by head or jaw movements. Additionally, participants with ST more often report fluctuations of their tinnitus and reaction to sleeping difficulties and low-intensity exercise. Large prospective cohort studies are needed to confirm these findings and to address the limitations of this study.

## Appendix

Multimedia Appendix 1 Survey questions.

Multimedia Appendix 2 Head and neck dysfunctions (in % of N=6115). TMD: Temporomandibular disorder.

Multimedia Appendix 3 Changes in tinnitus pitch or loudness (in % of N=6115).

Multimedia Appendix 4 Short-term effect of physical activity on tinnitus severity (in % of N=6115).

Multimedia Appendix 5 Presence of hearing-related characteristics in participants with and without somatosensory tinnitus.

Multimedia Appendix 6 Different tinnitus sounds in participants with and without somatosensory tinnitus.

Multimedia Appendix 7 Negative effects of stress, anxiety, and physical activity in participants with and without somatosensory tinnitus.

## Abbreviations

DC-ST: diagnostic criteria for somatosensory tinnitus
ST: somatosensory tinnitus
TMD: temporomandibular disorder
VAS: visual analogue scale
