# Supplementary material for: The Presence of Physical Symptoms in Patients With Tinnitus: International Web-Based Survey
Source: Interact J Med Res. 2019 Jul 30;8(3):e14519. doi: 10.2196/14519 (PMC6691675; doi:10.2196/14519)
Supplement: Multimedia Appendix 1 [file ijmr_v8i3e14519_app1.docx]

## Appendix 1 – Questionnaire

Q1 How long have you had tinnitus?

… months, … years

Q2 Are you...?

- Male
- Female
- Transgender
- Other / prefer to say

Q3 What is your age?

… years old

Q4 What country do you currently live in?

Q5 How loud would you rate your tinnitus?

0 1 2 3 4 5 6 7 8 9 10-

do not hear Extremely loud

Q6 What is the cause of your tinnitus? Please tell us any known causes of your tinnitus, in the order you believe them to be relevant - as many or as few as you are aware of.

Please indicate for each cause who diagnosed this cause:

- Doctor diagnosed
- Self-diagnosed
- Assumed

Q7 Does your tinnitus react to sound?

- Some sounds make it worse
- Some sounds make it worse but some make it better
- Some sounds can make it better
- No

Q8 Do you have any hearing loss?

- Mild hearing loss
- Moderate hearing loss
- Severe hearing loss
- Not diagnosed but I think I have hearing loss
- Not diagnosed but I don't think I have hearing loss
- No

Q9

How does your tinnitus behave during the day?

- Generally does not change in volume
- Generally grows louder as the day progresses
- Generally gets quieter as the day progresses
- Changes during the day or over days with no particular pattern

Q10 Do you get fleeting tinnitus episodes? Fleeting tinnitus is when your tinnitus loudness increases for a brief moment, your hearing may also become muffled.

- Daily
- Weekly
- Monthly
- A few times a year
- Less than a few times a year
- Never

Q11 Do you get a stiff neck or have sore neck muscles? Select all that apply.

- Yes, after certain physical activity
- Yes, from bad posture
- Yes, I have an associated medical condition
- Yes, from lying in bed / sleeping
- Yes, my neck movement is restricted due to stiffness
- No more than I believe is normal

Q12 Do you get headaches?

- Yes, they feel like they come from the neck
- Yes, they feel like they come from the jaw
- Yes, they come from both the neck and the jaw
- Yes, but I can't pinpoint a cause
- No more than I believe is normal

Q13 Do you have feelings of fullness in the ears? Select all that apply.

- Yes, after activity mainly
- Yes, after a bad sleep
- Yes, after listening to some sounds or being exposed to noise
- Yes, after working at a computer or desk
- Yes, after periods of stress / anxiety
- Yes, but I don't know what causes it
- No

Q14 Do you grind or clench your teeth?

- Yes, I grind them in my sleep
- Yes, I find that I often clench my teeth without realizing
- Both, I grind in my sleep and clench without realizing
- No, not that I am aware of

Q15 Does your tinnitus change with any of the below? Select all that apply. WARNING - if you perform any of the below your tinnitus may get louder.

- Pressing the jaw on the side
- Pushing the jaw backwards
- Pushing the lower jaw outwards rapidly
- Pushing your hand against your forehead while resisting with the neck muscles
- Clenching the teeth together
- Tilting your head backwards
- No change with any movements

Q16 Do you have pain or discomfort in the jaw? Select all that apply.

- Yes, my jaw sometimes feels painful
- Yes, I struggle to fully move my jaw
- Yes, my jaw sometimes feels tired
- Yes, muscles around my jaw feel tight or tense
- Yes, I have several popping and clicking noises in my jaw
- Yes, I have been diagnosed with TMJ dysfunction
- No real issues that I am aware of

Q17 How would you describe your tinnitus? Select all that apply.

- A pure tone / single pitch
- A mixture of tones
- A low buzzing
- A high buzzing
- Electric / interference type sound
- A low rumbling
- A static noise
- Clicking
- Intermittent beeping (morse code)
- A whooshing noise – pulsatile
- A whooshing noise - not in time with the pulse
- A pulsating sound
- Other (please specify)

Q18 How do the following affect your tinnitus?

Stress:

- A lot worse
- A little worse
- No difference
- A little better
- A lot better
- Unsure / don’t know

Anxiety:

- A lot worse
- A little worse
- No difference
- A little better
- A lot better
- Unsure / don’t know

Waking up from a good nights sleep:

- A lot worse
- A little worse
- No difference
- A little better
- A lot better
- Unsure / don’t know

Having a bad nights sleep:

- A lot worse
- A little worse
- No difference
- A little better
- A lot better
- Unsure / don’t know

Waking up from napping:

- A lot worse
- A little worse
- No difference
- A little better
- A lot better
- Unsure / don’t know

Intense workout (e.g. HiiT, weights, aerobics):

- A lot worse
- A little worse
- No difference
- A little better
- A lot better
- Unsure / don’t know

Moderate exercise (e.g. jogging, cardio):

- A lot worse
- A little worse
- No difference
- A little better
- A lot better
- Unsure / don’t know

Light exercise (e.g. walking, gardening):

- A lot worse
- A little worse
- No difference
- A little better
- A lot better
- Unsure / don’t know

Q19 Are you able to mask your tinnitus with sounds?

- Masked by nearly all sounds
- Masked by things such as TV, music or general background noise
- Masked by white noise or special masking sounds
- Masked only by a small selection of sounds
- Masked only in the shower or by other loud water type sounds
- Never masked

Q20 How often are you annoyed by your tinnitus?

Over the past week:

Never Always

0 1 2 3 4 5 6 7 8 9 10

On good days:

Never Always

0 1 2 3 4 5 6 7 8 9 10

On bad days:

Never Always

0 1 2 3 4 5 6 7 8 9 10

Right now:

Never Always

0 1 2 3 4 5 6 7 8 9 10

Q21 How aware have you been of your tinnitus over the past week? (on Visual analogue scale)
