# Supplementary figures and images for: The Presence of Physical Symptoms in Patients With Tinnitus: International Web-Based Survey
Source: Interact J Med Res. 2019 Jul 30;8(3):e14519. doi: 10.2196/14519 (PMC6691675; doi:10.2196/14519)

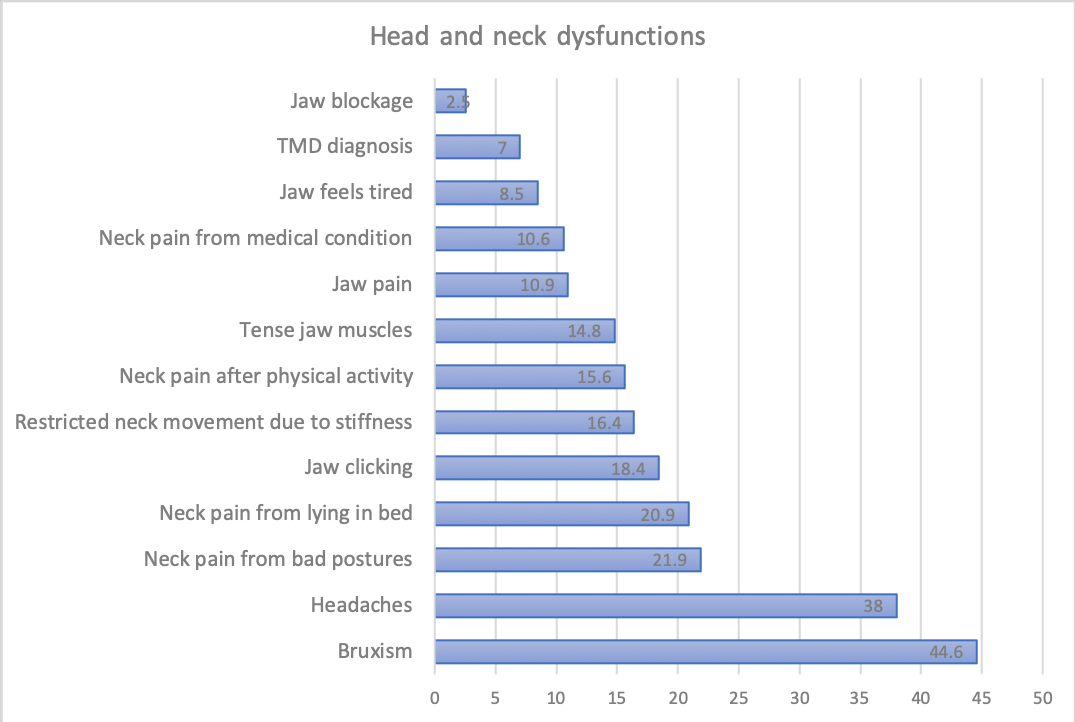

Supplement: Multimedia Appendix 2 [file ijmr_v8i3e14519_app2.png]

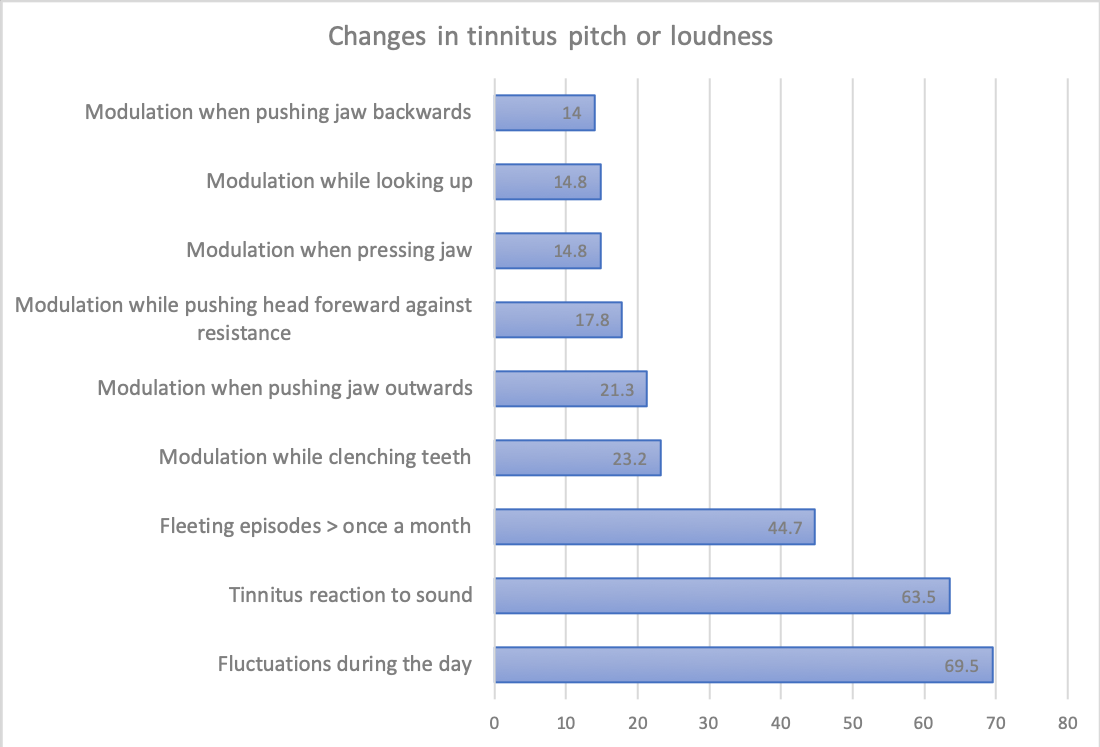

Supplement: Multimedia Appendix 3 [file ijmr_v8i3e14519_app3.png]

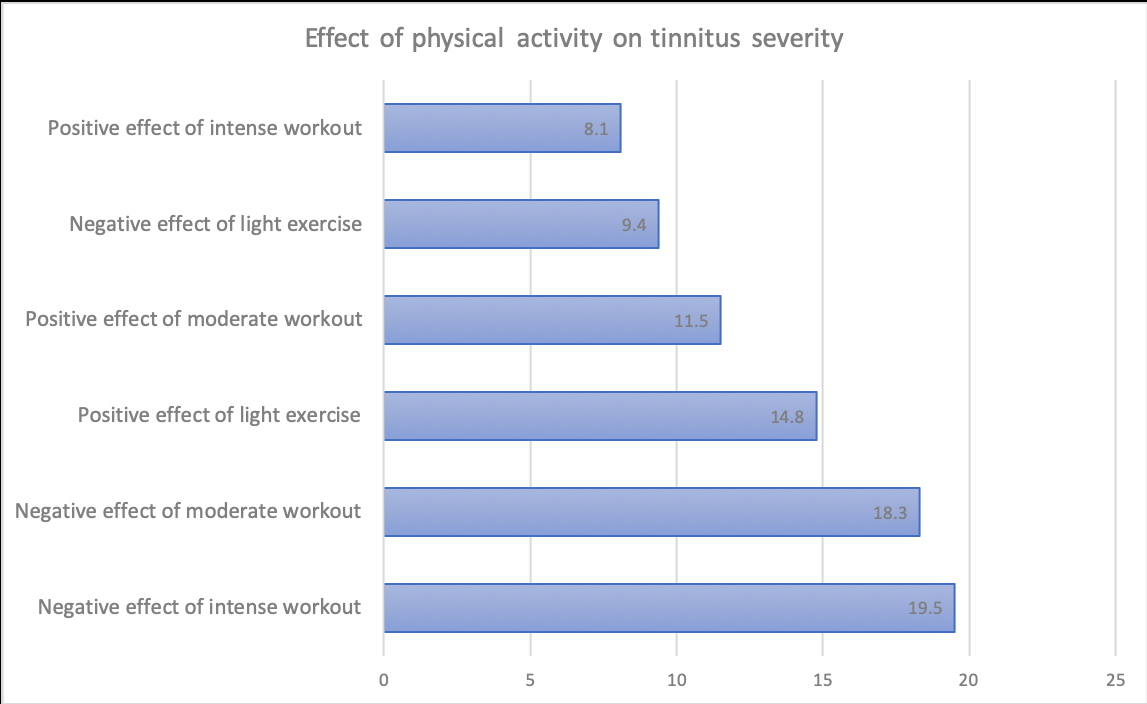

Supplement: Multimedia Appendix 4 [file ijmr_v8i3e14519_app4.png]
