# Supplementary material for: The Presence of Physical Symptoms in Patients With Tinnitus: International Web-Based Survey
Source: Interact J Med Res. 2019 Jul 30;8(3):e14519. doi: 10.2196/14519 (PMC6691675; doi:10.2196/14519)
Supplement: Multimedia Appendix 5 [file ijmr_v8i3e14519_app5.docx]

*Appendix 2: Presence of hearing related characteristics in participants with and without somatosensory tinnitus*

| Characteristics | ST-group %  (n=154) | non-ST group %  (n=1108) | Corrected p-value |
| --- | --- | --- | --- |
| Fullness in ears after activity | 9 | 4 | .033 |
| Fullness in ears after computer work | 6 | 3 | .126 |
| Fullness in ears after noise exposure | 16 | 12 | .128 |
| Fullness in ears after bad sleep | 10 | 6 | .192 |
| Fullness in ears without known cause | 45 | 39 | .224 |
| Fullness in ears after stress/anxiety | 18 | 14 | .376 |
| Hearing loss | 69 | 72 | .492 |
| Fleeting episodes > once a month | 69 | 72 | .493 |
| Tinnitus reaction to sound | 70 | 68 | .746 |

ST: somatosensory tinnitus
