# Supplementary material for: The Presence of Physical Symptoms in Patients With Tinnitus: International Web-Based Survey
Source: Interact J Med Res. 2019 Jul 30;8(3):e14519. doi: 10.2196/14519 (PMC6691675; doi:10.2196/14519)
Supplement: Multimedia Appendix 6 [file ijmr_v8i3e14519_app6.docx]

*Appendix 3: Different tinnitus sounds in participants with and without somatosensory tinnitus*

| Characteristics | ST-group %  (n=154) | non-ST group %  (n=1108) | Corrected p-value |
| --- | --- | --- | --- |
| Tinnitus pulsating sound | 12 | 8 | .089 |
| Tinnitus electric or interferance type sound | 27 | 20 | .091 |
| Tinnitus mixture of tones | 40 | 33 | .173 |
| Tinnitus high buzz | 40 | 34 | .173 |
| Tinnitus whoosh not in time with pulse | 8 | 7 | .492 |
| Tinnitus low buzz | 12 | 13 | .741 |
| Tinnitus clicking sound | 5 | 6 | .746 |
| Tinnitus whoosh noise (pulsatile) | 12 | 11 | .746 |
| Tinnitus pure tone | 36 | 37 | .823 |
| Tinnitus static noise | 19 | 18 | .894 |
| Tinnitus low rumbling | 5 | 5 | .921 |
| Tinnitus intermittent beeping | 5 | 5 | .975 |

ST : somatosensory tinnitus
