# Supplementary material for: The Presence of Physical Symptoms in Patients With Tinnitus: International Web-Based Survey
Source: Interact J Med Res. 2019 Jul 30;8(3):e14519. doi: 10.2196/14519 (PMC6691675; doi:10.2196/14519)
Supplement: Multimedia Appendix 7 [file ijmr_v8i3e14519_app7.docx]

*Appendix 4: Negative effects of stress, anxiety and physical activity in participants with and without somatosensory tinnitus*

| Characteristics | ST-group %  (n=154) | non-ST group %  (n=1108) | Corrected p-value |
| --- | --- | --- | --- |
| Negative effect of light exercise | 16 | 8 | .003 |
| Negative effect of bad nights sleep | 68 | 56 | .014 |
| Negative effect of stress | 77 | 68 | .052 |
| Negative effect of anxiety | 72 | 64 | .081 |
| Negative effect of intense workout | 25 | 20 | .327 |
| Negative effect of moderate exercise | 21 | 19 | .492 |

ST: somatosensory tinnitus
